# Association between blood-based protein biomarkers and brain MRI in the Alzheimer’s disease continuum: a systematic review

**DOI:** 10.1007/s00415-024-12674-w

**Published:** 2024-09-12

**Authors:** Micaela Mitolo, Gemma Lombardi, Riccardo Manca, Benedetta Nacmias, Annalena Venneri

**Affiliations:** 1 Department of Medicine and Surgery, University of Parma, Parma, Italy; 2 IRCCS Istituto delle Scienze Neurologiche di Bologna, Bologna, Italy; 3 Department of Neuroscience, Psychology, Drug Research and Child Health, University of Florence, Florence, Italy; 4 IRCCS Fondazione Don Carlo Gnocchi Onlus, Florence, Italy; 5 Department of Life Sciences, Brunel University London, Kingston Lane, Uxbridge, UB8 3PH UK

**Keywords:** Alzheimer’s disease, Blood-based biomarkers, MRI, Dementia, Mild cognitive impairment, Subjective cognitive decline

## Abstract

Blood-based biomarkers (BBM) are becoming easily detectable tools to reveal pathological changes in Alzheimer’s disease (AD). A comprehensive and up-to-date overview of the association between BBM and brain MRI parameters is not available. This systematic review aimed to summarize the literature on the associations between the main BBM and MRI markers across the clinical AD continuum. A systematic literature search was carried out on PubMed and Web of Science and a total of 33 articles were included. Hippocampal volume was positively correlated with Aβ42 and Aβ42/Aβ40 and negatively with Aβ40 plasma levels. P-tau181 and p-tau217 concentrations were negatively correlated with temporal grey matter volume and cortical thickness. NfL levels were negatively correlated with white matter microstructural integrity, whereas GFAP levels were positively correlated with myo-inositol values in the posterior cingulate cortex/precuneus. These findings highlight consistent associations between various BBM and brain MRI markers even in the pre-clinical and prodromal stages of AD. This suggests a possible advantage in combining multiple AD-related markers to improve accuracy of early diagnosis, prognosis, progression monitoring and treatment response.

**Supplementary Information:**

The online version contains supplementary material available at 10.1007/s00415-024-12674-w.

## Introduction

Timely and accurate diagnosis of Alzheimer’s disease (AD) in clinical practice is currently challenging, with misdiagnosis in the range of 20–25% when cerebrospinal fluid (CSF) or positron emission tomography (PET) biomarkers are not utilized [1, 2]. Therefore, suboptimal treatment and care, delayed or incorrect therapies, and inaccurate information about the disease and its prognosis are frequent.

Among the different biomarkers available for neurodegenerative diseases (NDDs), CSF-based biomarkers, which are predictive of brain pathological modifications, are formally integrated into the clinical diagnostic criteria for AD in their most recent formulation [3]. However, CSF sampling is invasive, expensive, and not suitable for screening purposes. Novel ultrasensitive methods, such as single-molecule array (Simoa) technology, allow the measurement of blood-based biomarkers (BBM) that show moderate to good accuracy in predicting amyloid status as assessed by either PET or CSF [4–6]. BBM have the potential advantage of being more accessible and cheaper than other established biomarkers, i.e. PET and CSF, and afford greater patient compliance. Moreover, BBM have been recently proposed as screening tools to detect AD in its earliest stages, before progression to AD dementia [7]. Beta-amyloid (Aβ) markers (e.g., Aβ42/40 ratio), phosphorylated tau (p-tau), neurofilament light chain (NfL), and glial fibrillary acidic protein (GFAP) are among the most advanced BBM for AD-relevant diagnostic and prognostic purposes [8], with p-tau showing high specificity and NfL having high sensitivity values. According to the biomarker classification proposed by Hampel et al. [9] and referred to as the “ATNX framework”, Aβ biomarkers belong to the “A” category, biomarkers of tau pathology (i.e., p-tau isoforms) to the “T” category, biomarkers of neurodegeneration or neuronal injury (e.g., t-tau and NfL) to the “N” category and GFAP and other BBM to the “X” category.

Not all BBM, however, have been found to perform equally well. Indeed, plasma Aβ42 peptides are highly labile and prone to aggregate, making their concentrations susceptible to variation in pre-analytical processing. The ratio of Aβ42/Aβ40 in plasma may be more useful than levels of individual Aβ peptides [10] for detecting abnormal Aβ status in both cognitively impaired [11, 12] and cognitively unimpaired people, even before Aβ positivity status can be detected by means of Amyloid PET (Aβ-PET) [13]. However, the plasma-based Aβ42/Aβ40 ratio may have lower diagnostic accuracy than the CSF-based Aβ42/Aβ40 ratio, thus representing a potential problem for its clinical application [8].

All plasma p-tau isoforms (i.e., p-tau181, p-tau217, p-tau231) have a high ability to discriminate AD from non-AD NDDs with high specificity [14–19]. Recent data have highlighted the promising performance of p-tau217 and p-tau231 in detecting AD before its clinical manifestations [17, 20–22].

T-tau is a biomarker of neurodegeneration that reflects the release of tau from neurons and non-specific changes in cortical thickness, but unrelated to neuronal loss [23]. T-tau levels increase in different tauopathies, such as frontotemporal dementia (FTD), corticobasal degeneration, and progressive supranuclear palsy. In the AD continuum, t-tau is often used in ratios with other biomarkers to improve its diagnostic specificity [24]. Given that blood NfL levels reflect neurodegeneration severity, but not the specific etiology, their assay is applicable to detect different NDDs, such as amyotrophic lateral sclerosis and atypical parkinsonisms. NfL levels have been suggested as a promising biomarker to discriminate cognitive decline due to AD in its prodromal or preclinical stages [25, 26], even if the validity of their discriminatory power has not been determined for levels of this biomarker obtained from blood [27]. This biomarker has also been suggested to have good prognostic value in NDDs such as Huntington's disease and Parkinson's disease [28].

GFAP levels in the blood, instead, reflect neuroinflammation and have been found to be higher in Aβ-positive than in Aβ-negative people, and in individuals with AD or mild cognitive impairment (MCI) than in healthy controls (HC) [29].

Furthermore, brain parameters obtained with multiple neuroimaging modalities, including magnetic resonance imaging (MRI) and fluorodeoxyglucose PET (FDG-PET), have provided possible markers of neurodegeneration with potential for clinical applications. Indeed, although neurodegeneration is a non-specific marker of AD in its biological definition [30], studies have shown that patients with evidence of neurodegeneration have a twofold increased risk of progression to dementia over a 5-year period than those without [31]. In this regard, structural MRI (sMRI), functional MRI (fMRI), diffusion tensor imaging (DTI) and magnetic resonance spectroscopy (MRS) can detect pre-symptomatic markers of neural alterations in cognitively unimpaired older adults and might also be used to monitor AD progression after its clinical onset [32], with more limited costs compared with other procedures [33].

The relationship between multiple biological and imaging markers has been largely studied to evaluate whether the combination of multiple biological variables improves accuracy of AD diagnosis and prediction of progression to AD dementia in cognitively unimpaired older adults and in patients with MCI.

Recently, many studies have been conducted to assess the association between CSF and MRI markers across the AD continuum, revealing a strong relationship for sMRI [34–38], DTI [39], fMRI [40, 41], and MRS [42].

In contrast, a comprehensive and up-to-date review on the association between BBM and brain MRI parameters is currently not available. Since the correlation between some blood- and CSF-based biomarkers is suboptimal, especially for Aβ42 [10, 43] and GFAP [44], it is possible that BBM may provide different and/or complementary insights on the neural alterations associated with AD in different disease stages. Indeed, the scientific literature on this topic is fast-growing. Simrén et al. [45] showed that plasma p-tau181 levels are increased in a subset of individuals with MCI and AD dementia, compared with HC, and are correlated with cognitive impairment and gray matter (GM) volume in temporal regions. Moreover, Verde et al. [18] provided a short review on the association between p-tau isoforms and neuroimaging features, highlighting that plasma p-tau181 levels are negatively correlated (mainly in Aβ-positive individuals) with whole brain volume as well as GM volumes of several temporo-parietal areas (i.e., hippocampus, entorhinal cortex, precuneus, and posterior cingulate cortex), cortical thickness of the temporal lobe and of an AD-signature region, and fractional anisotropy (FA) values in the genu of the corpus callosum. However, these results have been reported without details relative to the cognitive status of the participants included in the reviewed studies. Therefore, the aim of the present review was to expand the currently available knowledge on the association between the most established BBM of AD (i.e., Aβ40, Aβ42/40 ratio, Aβ42, p-tau, NfL, GFAP) and brain MRI parameters along the AD continuum.

## Materials and methods

### Search strategy

The review was carried out following the Preferred Reporting Items for Systematic Review and Meta-Analysis (PRISMA) guidelines [46]. A systematic literature search was carried independently by three authors (MM, GL and RM) in May 2023 using two databases, PubMed and Web of Science; any discrepancies during the screening process were resolved through discussion. The following search terms were used: “blood biomarker” or “plasma” or “serum” combined with “Alzheimer’s disease” or “AD” or “Mild Cognitive Impairment” or “MCI” or “Subjective cognitive decline” or “SCD” and “structural MRI” or “functional MRI” or “diffusion tensor imaging” or “MRS” or “Magnetic Resonance Spectroscopy”. In addition, the reference lists of the selected original articles/reviews on similar topics were searched for additional eligible records.

### Study eligibility criteria

Studies were considered eligible if they assessed the relationship between brain MRI parameters and at least one of the following main peripheral BBM, i.e., Aβ, p-tau, t-tau, NfL and GFAP, derived from patients across the AD continuum, from Subjective Cognitive Decline (SCD) to AD dementia. The following exclusion criteria were defined to identify all relevant studies: (1) no data on the selected blood-based protein biomarkers; (2) no correlation reported between blood biomarkers and MRI data; (3) meta-analysis, review articles or study protocols; (4) studies that included only cognitively unimpaired older adults; (5) studies that included patients with other neurological conditions; (6) animal studies; (7) molecular imaging studies; (8) non-peer reviewed articles; (9) articles written not in English; (10) case reports. Year of publication was not considered as inclusion/exclusion criterion, as we aimed to capture all existing research in this field.

### Study selection

**Fig. 1:**
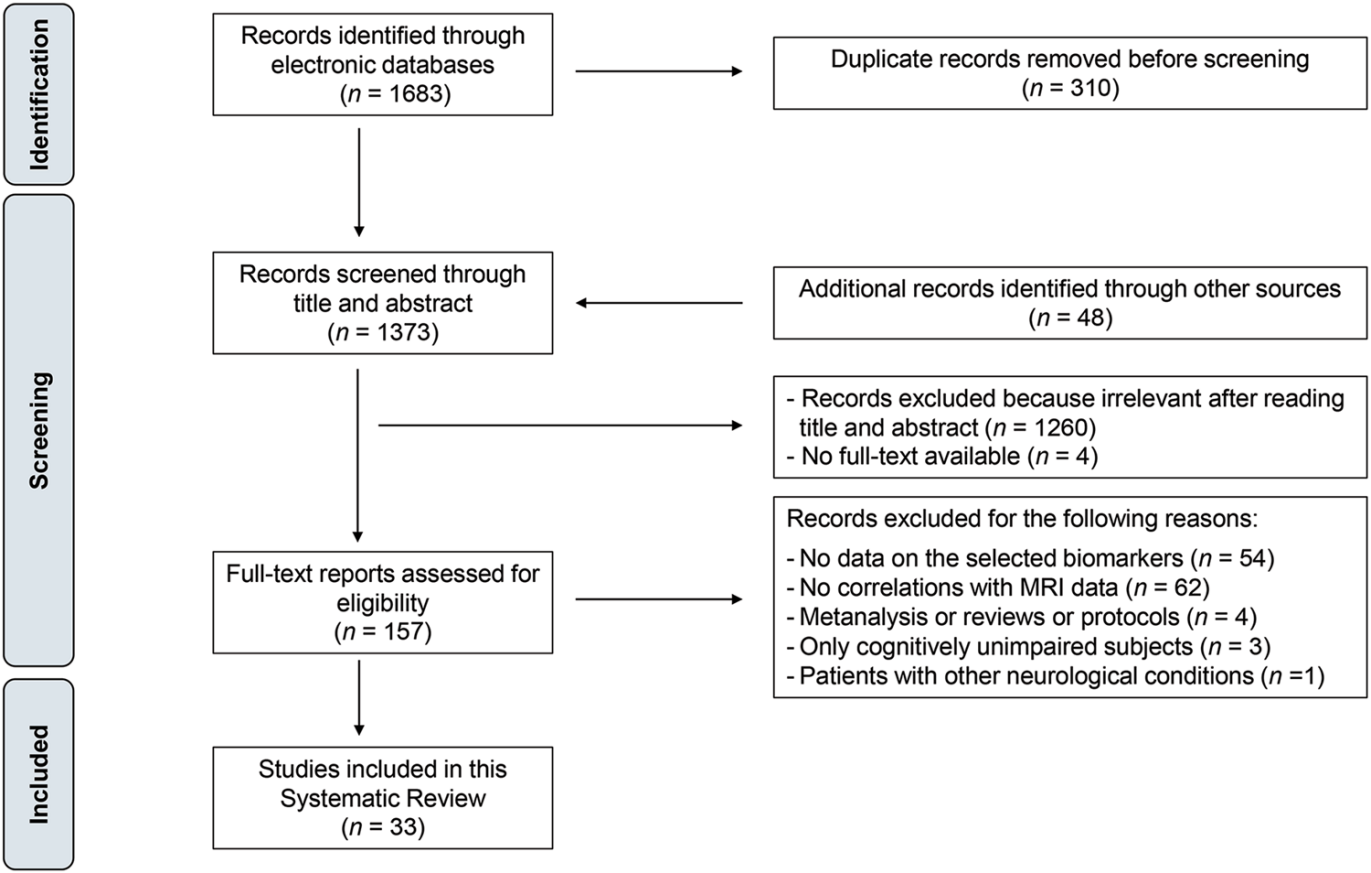
PRISMA flowchart describing the study selection process

The initial literature search produced a total of 1683 records of which 310 were duplicate publications that were found in both the PubMed and Web of Science databases. Following removal of duplicates, 1373 records were screened by title and abstract; in addition, 48 more records were identified through other sources (i.e., the reference lists of selected original articles and papers/reviews on a similar topic) and added to the screening process. A total of 157 full-text reports were retrieved and assessed for eligibility. Based on the inclusion/exclusion criteria, 124 articles were excluded for the following reasons: 54 studies did not include data on the selected blood-based protein biomarkers; 62 did not report correlations between BBM and MRI data; 4 reported data from meta-analyses, review articles or study protocols; 3 studies included only cognitively unimpaired older adults; and one study focused on patients with other neurological conditions. Therefore, 33 unique studies were included in the systematic review. The study selection process is described in Fig. 1.

We have described all studies according to the “ATNX” framework by Hampel and colleagues [9] that suggested the addition of an “X”, inflammatory markers, to the ATN biomarker framework to reflect the whole continuum of AD.

### Quality assessment

To the best of our knowledge, no standard tool to assess the quality of studies using MRI and/or blood-biomarkers for AD is available to date. Therefore, a customized scale (Table S1) was created for this study and included 15 assessment criteria to evaluate 5 areas (i.e., case definition, general methods, biomarker methods, MRI methods and reporting of findings). A total score was generated for each study as a percentage of the maximum score (i.e., 18).

## Results

Details on demographic characteristics, BBM and MRI techniques used, and the main findings of each study are summarized in Table 1. All the 33 studies included in this systematic review were published between 2006 and 2023 and assessed the association between BBM (i.e., Aβ, p-tau, t-tau, NfL and GFAP) and brain parameters derived from structural MRI, DTI, and MRS.

**Table 1:** Summary of the characteristics and main findings of the 33 studies included in the systematic review

Authors (year)	Sample (*N*)	Gender (M %)	Age M (SD)	BBM (technique)	MRI technique	Statistical approach	Key findings	Study quality (%)
Altomare et al. (2023)	SCD (82) MCI (99) AD dementia (19)	44% 53% 53%	71 (12) 73 (10) 76 (8)	NfL (Simoa HD-X Analyzer)	Structural MRI (hippocampal volume)	Pearson correlation test	Plasma NfL levels were negatively correlated with hippocampal volume in the whole sample	58.8
Asken et al. (2022)	Cohort 1: HC (39) MCI (11) Cohort 2: HC (32) MCI (18) AD dementia (21)	Cohort 1: 48% Cohort 2: 49%	72.7 (6.3) 70.7 (8.6) 75.4 (4.6) 70.4 (11.2) 68.9 (11.2)	NfL; GFAP (Simoa on the same HD-1 Analyzer for both cohorts)	Structural MRI (ROI volumes)	Linear regression model Covariates: plasma GFAP, plasma NfL, age, gender, TIV	Cohort 2: NfL levels were negatively associated with parietal GM volume NfL levels were not associated with GM or WM volumes after adjusting for plasma GFAP levels Both Cohorts: GFAP levels were negatively associated with temporal and parietal WM volumes	61.1
Barker et al. (2021)	HC (51) AD dementia (156)	21% 43%	70.8 (5.9) 74.8 (8.2)	NfL (Simoa HD-1 Analyzer)	Structural MRI (hippocampal volume/TIV)	Pearson correlation test	NfL levels were negatively associated with hippocampal volume/TIV ratio in the AD dementia group only	52.9
Benedet et al. (2020)	HC (382) CI (767) composed by: MCI (420); AD dementia (347)	45% 59%	73.5 (6.9) 74.4 (7.8)	NfL (Simoa HD-1 Analyzer)	Structural MRI (VBM)	Linear model Covariates: age, sex, diagnosis, APOE, years of education	NfL levels were negatively associated with GM and WM volumes in both groups Associations were found in PCC, frontal and temporal cortices in the HC group, only in ApoE ε4 carriers; More widespread associations were found in the CI group	77.8
Cantero et al. (2016)	HC (48) SCD (47)	54% 46%	68.1 (3.2) 69.6 (4.3)	Aβ42 (ELISA)	Structural MRI (hippocampal subregions)	Regression analysis adjusted for age and TIV	Aβ42 levels were negatively associated with volume of DG and of the molecular layer in the SCD group	72.2
Ebenau et al. (2022)	SCD (401)	42%	60.9 (8.5)	NfL; GFAP (Simoa GFAP Discovery Kit Quanterix and Simoa NF-Light Advantage Kit Quanterix)	Structural MRI (hippocampal volume; MTA)	Pearson correlation test and partial correlation adjusted for age and sex	NfL and GFAP levels were negatively associated with hippocampal volume and positively with MTA (weak correlation, even weaker after adjustment for age and sex)	58.8
Elahi et al. (2020)	HC (33) EOAD (33) LOAD (30)	67% 64% 70%	72 (8.4) 61 (6.2) 79 (4.7)	NfL; GFAP (Simoa HD-1 Analyzer)	Structural MRI (GMV, WMH)	Regression analysis adjusted for age and TIV	No significant associations were found between BBM and GMV, whereas both BBM were positively associated with the WMH volume in the whole sample	58.8
Fan et al (2018)	HC (39) MCI (25) AD dementia (16)	41% 56% 56%	63 (8.5) 68 (9.8) 67.6 (12.2)	Aβ40; Aβ42; t-tau (antibodies used for the reagent plus immunomagnetic reduction assay)	Structural MRI (mCT; GM and WM volumes)	Spearman correlation analysis	Aβ40 levels were positively correlated with mCT Aβ42 and t-tau levels were negatively correlated with mCT (correlation of Aβ42 only in Aβ-PET negative participants)	52.9
Gurol et al. (2006)	MCI (18) AD dementia (36) CAA (42)	56% 50% 48%	73 (8.5) 77.7 (7.5) 73.9 (6.8)	Aβ40; Aβ42 (ELISA)	Structural MRI (WMH volumes/TIV)	Partial correlation and regression model (effect modification of Aβ by APOE genotype or diagnosis examined by interaction terms)	Aβ40 levels were positively associated with larger WMH volume in all groups	41.2
Hanon et al. (2018)	naMCI (122) aMCI (417) AD dementia (501)	28% 45% 43%	77.9 (5.2) 77.7 (5.5) 77.3 (7.7)	Aβ40; Aβ42 (INNO-BIA kit Fujirebio based on a multiplex xMAP technique with a LABScan-200 system Luminex)	Structural MRI (hippocampal volume)	Pearson correlation test	Aβ40 levels were negatively correlated with hippocampal volume in the AD dementia group No correlations were found between Aβ42 levels and hippocampal volume	52.9
Hsu et al (2017)	HC (108) MCI (60) AD dementia (177)	56% 55% 51%	74.6 (7.7) 73.9 (8.3) 81 (5.8)	Aβ40; Aβ42; Aβ42/Aβ40 ratio (INNO-BIA kit Fujirebio based on a multiplex xMAP technique with a LABScan-200 system Luminex)	Structural MRI (visual rating MTA and PA)	Regression analysis Covariates: age, years of education, gender, APOE and Pearson correlation test	Aβ42/Aβ40 ratio was negatively associated with the mean MTA score in the AD dementia group	55.6
Illan-Gala et al. (2021)	HC (55) AD dementia (43)	45% 37%	52.2 (13) 65.2 (10)	NfL; t-tau (Simoa HD-1 Analyzer)	Structural MRI (cortical thickness)	Multiple regression analysis Covariates: age, gender	NfL levels were negatively correlated with right lateral temporal lobe, right inferior parietal, and left superior frontal cortical thickness values in the AD dementia group t-tau levels were not associated with cortical thickness in the AD dementia group	66.7
Karikari et al. (2020)	Young HC (27) Old HC (113) MCI (45) AD dementia (33)	37% 36% 49% 55%	22.7 (1.9) 69.2 (9.7) 72.6 (6.8) 64.6 (9.2)	p-tau181 (Simoa HD-1 Analyzer)	Structural MRI (hippocampal volume)	Linear regression model Covariates: age, gender, APOE, years of education	P-tau181 levels were negatively correlated with hippocampal volume in the AD dementia group only	66.7
Karikari et al. (2021)	HC Aβ- (268) HC Aβ + (68) MCI Aβ- (277) MCI Aβ + (209) AD dementia Aβ- (41) AD dementia Aβ + (137)	51% 37% 55% 59% 78% 52%	73.5 (6.5) 76.9 (6.2) 71.4 (8) 73.9 (6.7) 77.3 (7) 73.4 (8.2)	p-tau181 (Simoa HD-X Analyzer)	Structural MRI (hippocampal, ventricular and whole brain volumes)	Linear regression model adjusted for age and gender	P-tau181 levels were negatively correlated with hippocampal and total brain volumes and positively correlated with ventricular volume in the whole group In regression analyses stratified by diagnostic group, a significant association was found between p-tau181 levels and hippocampal volume in HC and MCI only	55.6
Krebs et al. (2023)	SCD (52)	42%	71 (5.7)	Aβ42/Aβ40 ratio; p-tau181 (Simoa HD-1 Analyzer)	Structural MRI (hippocampal atrophy calculated as: 1 − hippocampal volume/TIV)	Partial correlation analysis controlling for age	Negative associations were found between Aβ42/40 ratio values and hippocampal atrophy No significant correlation was found between p-tau181 levels and hippocampal atrophy	66.7
Marks et al. (2021)	HC (864) MCI (131) Mayo cohort HC (190) MCI (107) ADNI cohort	55% 60%	75.6 81.4	NfL; t-tau (Simoa HD-1 Analyzer)	Structural MRI (hippocampal volume/TIV, cortical thickness, WMH); DTI (corpus callosum FA)	Linear mixed effects model adjusted for age, gender, education, previous exposure to cognitive battery	NfL levels were associated with hippocampal volume in the ADNI cohort Elevated plasma t-tau was associated with lower thickness of temporal region in the Mayo cohort	61.1
Mattson et al. (2016)	HC (189) MCI (195) AD (179) ADNI cohort	55% 67% 52%	75.9 (4.9) 74.7 (7.5) 75.2 (7.4)	t-tau (Simoa HD-1 Analyzer)	Structural MRI (ventricular volume, hippocampal volume)	Linear mixed effects model adjusted for age, gender, diagnostic group, APOE, years of education, TIV	T-tau levels were negatively associated with ventricular volume	64.7
Mattson et al. (2017)	HC (193) MCI (197) AD dementia (180)	55% 67% 51%	75.9 (4.9) 74.7 (7.5) 75.3 (7.3)	NfL (Simoa HD-1 Analyzer)	Structural MRI (ventricular and hippocampal volume, cortical thickness, WMH)	Linear mixed effects model adjusted for age, gender, diagnostic group, APOE, years of education, TIV	NfL levels were negatively associated with ventricular and hippocampal volume, and with thinner cortices in an AD-related cortical ROI in the whole sample (stronger correlations in MCI and AD dementia than HC)	61.1
Mielke et al. (2021)	HC Aβ- (89) HC Aβ + (88) MCI Aβ- (10) MCI Aβ + (13)	56% 44% 70% 38%	76.4 81.8 71.6 84.8	p-tau181 (Simoa) p-tau231 (Simoa) p-tau217 (MSD) p-tau 181 (MSD) Simoa HD-X Analyzer for p-tau 181, Simoa in-house method for p-tau 231 MSD method developed by Lilly	Structural MRI (temporal meta-ROI cortical thickness, WMH) DTI (FA values in the genu of the corpus callosum and in the HCB)	Linear regression model adjusted for age, gender, APOE, years of education, BMI, CKD	All p-tau measures were negatively associated with temporal lobe cortical thickness Levels of Simoa p-tau181, MSD p-tau181, and MSD p-tau217, but not of Simoa p-tau231, were positively associated with WMH volumes and negatively with FA values in the genu of the corpus callosum FA values in the HCB were negatively associated with MSD p-tau217 levels only	55.6
Nabizadeh et al. (2022a)	MCI (92)	55%	73.04 (6.4)	NfL (Simoa)	DTI (voxel-based analysis of FA, AxD, MD, RD indices)	Linear regression model stratifying for APOE, age, gender	NfL levels were negatively associated with FA and positively with RD, AxD, and MD values in WM tracts that differed between APOE ε4 carriers (primarily the internal capsule and the corona radiata) and non-carriers (primarily the cingulum bundle)	50.0
Nabizadeh et al. (2022b)	HC (43) MCI (119) AD dementia (41)	51% 59% 58%	72.9 (6.2) 72.8 (6.8) 74 (8.6)	p-tau181 (Simoa technique by two monoclonal antibodies (Tau12 and AT270) which test N-terminal to mid-domain forms of p-tau181 HD-1)	DTI (voxel-based analysis of FA, AxD, MD, RD indices)	Linear regression model controlled for education, FDG PET metabolism in parietal-temporal regions, MMSE, APOE	P-tau181 levels were positively correlated with MD, RD, AxD, and negatively with FA values in different WM regions across groups: Left primarily medial lemniscus, fornix and hippocampal cingulum in the AD dementia group Right corona radiata, internal capsule and tapetum in the MCI group Left uncinate fasciculus and right fornix, sagittal stratum and cerebellar pedunculi in the HC group	55.6
Ossenkoppele et al. (2021)	HC (219) MCI/ AD dementia (181)	53% 50%	64.6(12.8) 71.5 (7.8)	p-tau181 p-tau217 (Simoa HD1 Analyzer for p-tau181, MSD platform for p-tau217)	Structural MRI (hippocampal volume/TIV, AD-signature cortical ROI thickness)	Pearson correlation test and ridge regression model adjusted for age and gender	Both p-tau isoforms were negatively correlated with an AD-signature cortical ROI thickness (i.e. bilateral entorhinal, inferior and middle temporal and fusiform cortices) in the whole sample	66.7
Parbo et al. (2020)	MCI/ AD dementia (27)	70%	73.6 (6.1)	NfL (Simoa)	Structural MRI (cortical thickness); DTI	Linear regression model	NfL levels were negatively correlated with cortical thickness of a cluster in the left frontal lobe NfL levels were positively correlated with MD values in areas of the temporal lobes and the cingulate cortex in the whole sample	44.4
Pereira et al. (2017)	HC Aβ- (57) HC Aβ + (37) MCI Aβ- (36) MCI Aβ + (109) AD dementia Aβ- (5) AD dementia Aβ + (65)	47% 57% 67% 61% 80% 52%	74.8 (5.2) 76.5 (5.2) 74.5 (9) 74.2 (6.9) 82.2 (5.4) 73.7 (7.6)	NfL (Simoa)	Structural MRI (volumes and cortical thickness)	Partial correlation analysis controlling for age, gender and diagnostic group	In the whole sample, NfL levels were negatively correlated with volumes of the hippocampus and nucleus accumbens NfL levels were negatively correlated with volumes of the hippocampus and nucleus accumbens in the MCI group only, independently of amyloid status In the whole sample, NfL levels were negatively correlated with cortical thickness in the left precuneus and right middle temporal gyrus NfL levels were negatively correlated with cortical thickness in different regions across groups The right precuneus and fronto-parietal areas in AD dementia Aβ+ Left precuneus and right superior parietal areas in MCI Aβ+ Left fronto-temporal and right fronto-insular-parietal areas in MCI Aβ–	77.8
Poljak et al. 2016	HC (129) aMCI (93) AD dementia (37)	46%	77.3 (4.7) 78.2–79.5 (4.8–4.9) 75.4 (7.7)	Aβ40; Aβ42; Aβ42/Aβ40 ratio (ELISA)	Structural MRI (hippocampal volumes, WMH)	Ordinary Least Squares (OLS) regression analysis controlling for age, gender, APOE, TIV	Aβ42 levels and the Aβ42/40 ratio were positively associated with hippocampal volume; Aβ42 levels were also negatively associated with WMH volume (differential associations of Aβ40 and Aβ42 emerged in ApoE ε4 carriers and non-carriers)	66.7
Schultz et al. (2020)	NCs (84) MCs (117: 87 PSEN1, 12 PSEN2, and 18 APP)	42% 50%	40.5 (10.7) 38.6 (10.8)	NfL (Simoa HD-1 Analyzer)	DTI (WM ROIs and voxel-based analysis of FA, AxD, MD, RD indices); WMH	Linear mixed effects model Covariates: age, gender	In the MC cohort, NfL levels were positively associated with total WMH volume and MD, AxD and RD values and negatively with FA values across all WM tracts investigated, apart from the corticospinal tract In the NC group, no associations between NfL levels and DTI parameters were found	83.3
Shahid et al. (2022)	HC (47) MCI (52) AD dementia (19)	23% 42% 42%	70.7 (4.8) 72.9 (6.6) 72.8 (8.4)	NfL; t-tau; Aβ40; Aβ42; Aβ42/Aβ40 ratio (Simoa HD-1 Analyzer)	DTI (microstructural alteration in the hippocampal subregions)	Partial correlation analysis accounting for age, gender, years of education, APOE, TIV	In the whole sample and in the HC group, only NfL was positively associated with microstructural parameters in CA4-DG In the AD dementia group, t-tau level was negatively associated with microstructural parameters in the subiculum and CA4-DG No significant associations were found in the MCI group Levels of Aβ40, Aβ42, Aβ42/Aβ40 were not associated with microstructural parameters	66.7
Shir et al. (2022)	HC (177) MCI (23) Aβ-PET- (99) Aβ-PET + (101)	58% 44%	75 (9)	GFAP (Quanterix Simoa HD-X Analyzer using the Simoa Neurology 4-Plex E Advantage kit)	Structural MRI (temporal meta-ROI cortical thickness; WMH)	Spearman correlation analysis and linear regression model adjusted for age and gender	GFAP levels were negatively associated with temporal meta-ROI cortical thickness and greater WMH volume in the Aβ-PET + group only	64.7
Sotolongo-Grau et al. (2014)	HC (49) MCI (33) AD dementia (14)	26% 27% 29%	56.2 (5.6) 74.6 (6.4) 79.5 (5.3)	DA, TP, CP Aβ40 DA, TP, CP Aβ42 (ELISA kit for the three blood fractions)	Structural MRI (Freesurfer ROIs)	Partial correlation analysis adjusted for age, gender, APOE, CKD	In all diagnostic group, Aβ40 CP levels were negatively correlated with mCT and left hippocampal and left entorhinal cortex volumes	44.4
Spotorno et al. (2022)	HC Aβ- (259) HC Aβ + (79) MCI Aβ + (90)	44% 42% 52%	67 (10) 73 (9) 73 (7)	GFAP (Simoa HD-X Analyzer)	MRS in the PCC/precuneus	Linear regression model Covariates: age, gender, cognitive status	GFAP levels were positively associated with mIns/tCr values in the PCC/precuneus in ApoE ε4 carriers	64.7
Thijssen et al. (2021)	MCI (99; 87 with MRI data) separately analysed	56%	65.5 (13)	p-tau217 p-tau181 (SULFO-TAG-Ru-4G10-E2 anti-tau monoclonal antibody)	Structural (GM atrophy)	Voxel-wise correlation analysis	Levels of p-tau217 and p-tau181 were negatively associated with temporo-parietal GM volumes, more strongly in Aβ-PET + participants	77.8
Wang et al. (2021)	SCD with low Aβ40 and Aβ42 (71) SCD with high Aβ40 and Aβ42 (71)	32% 31%	65.7 (3.64) 66.45 (4.1)	Aβ40; Aβ42 (MSD)	DTI (TBSS)	Voxel-wise correlation analysis corrected for age, gender, years of education	In the SCD group, Aβ40 levels were negatively associated with FA and positively with MD values in widespread WM tracts No significant correlations were found between Aβ42 and FA/MD values These results persisted after removing participants with vascular comorbidities	66.7
Weston et al. (2017)	NC (11) MC (37 PSEN1/APP: 19 pre-symptomatic, 18 symptomatic)	27% 53% 72%	38.9 (9.5) 36 (5.7) 46.6 (9.3)	NfL (Simoa HD-1 Analyzer)	Structural MRI (whole brain, ventricular, and hippocampal volumes)	Spearman correlation analysis	In all MC participants, NfL levels were negatively correlated with whole brain and hippocampal volumes and positively with ventricular volume	61.1

*Aβ* amyloid beta, *Aβ-PET* amyloid PET, *AD* Alzheimer’s disease, *aMCI* amnestic mild cognitive impairment, *APOE* apolipoprotein E, *APP* amyloid precursor protein, *AxD* axial diffusivity, *BBM* blood-based biomarkers, *BMI* body mass index, *CA* cornu ammonis, *CAA* cerebral amyloid angiopathy, *CKD* chronic kidney disease, *CI* cognitive impairment, *CP* cellular pellet, *CSF* cerebrospinal fluid, *DA* directly accessible, *DG* dentate gyrus, *DR* radial diffusivity, *DTI* diffusion tensor imaging, *EOAD* early onset Alzheimer’s disease, *FA* fractional anisotropy, *GFAP* glial fibrillary acidic protein, *GM* grey matter, *HC* healthy control, *HCB* hippocampal cingulum bundle, *LOAD* late onset Alzheimer’s disease, *M* males, *MC* mutation carrier, *MCI* mild cognitive impairment, *mCT* mean cortical thickness, *MD* mean diffusivity, *mIns* myo-inositol, *MRI* magnetic resonance imaging, *MRS* magnetic resonance spectroscopy, *MSD* meso-scale discovery, *MTA* medio-temporal atrophy, *naMCI* non-amnestic mild cognitive impairment, *NC* non-carrier, *NfL* neurofilament light, *p-tau* phosphorylated tau, *PA* parietal atrophy, *PCC* posterior cingulate cortex, *PSEN1/2* presenilin 1/1, *ROI* region of interest, *SCD* subjective cognitive decline, *t-tau* total tau, *TBSS* tract-based spatial statistics, *tCr* total creatine, *TIV* total intracranial volume, *TP* total in plasma, *WM* white matter, *WMH* white matter hyperintensity

A total of 10 studies assessed the “A” (i.e., Aβ40, Aβ42 and Aβ42/Aβ40 ratio), 7 focused on the “T” (i.e., p-tau181), 17 studies focused on the “N” (i.e., NfL, t-tau) and 5 assessed the “X” (i.e., GFAP) within the ATNX diagnostic framework of AD.

The BBM analytical procedures used in the reviewed studies were as follows: 24 studies used the Simoa technique [47] that allows quantification down to subfemtomolar concentrations (< 1 pg/mL), including Simoa with HD-1 ultrasensitive Analyzer by Quanterix, and the new flagship HD-X, i.e., the latest model fully automated bead-based immunoassay platform; three studies used the Meso Scale Discovery (MSD) platforms that are more sensitive and require less sample volume than the conventional ELISA kit [48], whereas the ELISA kit was used in 4 studies to assess Aβ isoforms; in 2 studies [49, 50] Aβ isoforms were assessed using the INNO-BIA kit (Fujirebio) based on a multiplex xMAP technique with a LABScan-200 system (Luminex), a technique with recognized good analytical performance and clinical sensitivity [49]; in 2 studies, BBM were quantified with both Simoa and MSD methods [51, 52], but only Mielke et al. [51] applied the 2 methods to assess plasma concentrations of the same biomarkers (i.e., p-tau181), thus enabling inter-method agreement assessment.

The MRI techniques used included: 27 studies used structural MRI, and the majority of these studies assessed the association between BBM and regional volumes; hippocampal volume was the MRI outcome measure most commonly investigated (15 studies), whereas 9 studies assessed the association between BBM and cortical thickness of different brain regions; 7 studies evaluated the relationship between DTI parameters of white matter (WM) microstructural integrity and BBM, and only one study assessed the relationship between MRS indices and BBM [53]; no studies investigated associations between BBM and fMRI parameters. The findings of this review are represented visually in Fig. S1.

### Detailed results of “A” biomarkers

Of the 10 studies that assessed the association between Aβ biomarkers (i.e., Aβ40, Aβ42 and Aβ42/Aβ40 ratio) with brain MRI parameters, most of them used structural MRI techniques. Four of these focused their analysis on either hippocampal subregions or on the whole hippocampal volume. In older adults with SCD, higher Aβ42 levels were associated with smaller volume of the dentate gyrus and of the molecular layer [33]; the plasma Aβ42/40 ratio was negatively associated with hippocampal atrophy [54] and, in people with AD dementia, with a medial temporal atrophy (MTA) score [50]. In contrast, Hanon and colleagues [49] found no significant correlation between hippocampal volume and plasma Aβ42 levels in people with symptomatic AD (i.e., with either MCI or dementia), but a weak negative correlation with plasma Aβ40 levels in the AD dementia group only. In the Sydney Memory and Ageing Study of Poljak et al. [55], plasma Aβ42 levels and the Aβ42/Aβ40 ratio were positively associated with hippocampal volume across the AD continuum, while Aβ42 was also negatively associated with white matter hyperintensity (WMH) volume. After stratification by ApoE genotype, different patterns of association were detected for the Aβ isoforms: Aβ40 was negatively correlated with hippocampal volume only in the ApoE ε4 carriers, whereas Aβ42 and Aβ42/Aβ40 ratio were positively correlated with hippocampal volume in the ApoE ε4 carriers and negatively with WMH volume in the ApoE ε4 non-carriers. An older study, however, had found that plasma levels of Aβ40 were positively associated with larger WMH volume in a mixed sample of AD dementia, MCI and cerebral amyloid angiopathy cases [56].

Two studies investigated the mean cortical thickness (mCT) of all cortical regions in people across the AD continuum (i.e., HC, MCI and AD dementia). Fan and colleagues [57] found that plasma Aβ42 levels were negatively correlated with mCT in Aβ-PET negative people, while Aβ40 levels were positively correlated with mCT in both Aβ-PET positive and negative participants. Sotolongo-Grau et al. [58] assessed plasma Aβ markers considering both total plasma Aβ pool and the peptide associated with the cellular pellet (CP): the strongest (negative) association was detected between Aβ40 CP levels and the left hippocampal volume and the left entorhinal cortex, but also with mCT values; Aβ42 CP levels, instead, were negatively correlated with hippocampal volume only.

Moreover, 2 studies investigated the association between Aβ and DTI indices. Shahid and colleagues [59] explored the associations of diffusion microstructural metrics in the hippocampal subfields with different plasma biomarkers of AD pathology and they found no significant associations between any microstructural parameters and either Aβ40, Aβ42, or Aβ42/Aβ40 ratio in a sample comprising HC, MCI, and AD dementia groups. Instead, Wang and colleagues [60] using a Tract-Based Spatial Statistics approach found significant associations between high plasma Aβ40 levels and microstructural parameters, specifically low FA and high MD values in widespread WM tracts in a group of people with SCD.

### Detailed results of “T” biomarkers

A total of 7 studies assessed the associations between p-tau isoforms, especially p-tau181, and brain MRI parameters. Two studies from the same research group on different cohorts found that higher plasma p-tau181 levels were negatively correlated with hippocampal volume only in AD dementia patients [61]. The second study found that, across the AD continuum, there was a negative correlation with total brain volume and a positive correlation with ventricular volume [62]. In the latter study, after stratifying by diagnostic group and accounting for age and sex differences, higher p-tau181 levels were associated with lower hippocampal volume in the HC and MCI groups only, but not in patients at the dementia stage. In contrast, Krebs et al. [54] found no significant associations between p-tau181 levels and brain MRI parameters in a group of people with SCD. Moreover, one study that investigated WM microstructural parameters found that plasma p-tau181 levels were positively correlated with mean diffusivity (MD), radial diffusivity (RD), axial diffusivity (AxD), but negatively correlated with FA values in different WM tracts across diagnostic groups [63]. In detail, p-tau181 levels were primarily associated with FA and AxD values in left-sided limbic WM connections (i.e., hippocampal cingulum, fornix and lemniscus) in the AD dementia group, and with all DTI indices in right-sided associative and projection tracts (i.e., tapetum, posterior corona radiata and the retrolenticular part of the right internal capsule).

Only a few studies assessed multiple p-tau isoforms. Ossenkoppele et al. [52] found associations between MRI parameters and p-tau217 and p-tau181 plasma levels: both p-tau measures were negatively correlated with cortical thickness values of an AD-signature region of interest comprising bilateral entorhinal, inferior and middle temporal and fusiform cortex in a mixed group of HC, MCI and AD dementia. Similarly, both p-tau217 and p-tau181 plasma levels were found to be associated with lower temporo-parietal GM volume in Aβ-PET positive patients with MCI [64]. In addition, Mielke et al. [51] showed that, in a group comprehensive of HC and MCI patients, increasing levels of Simoa p-tau181, MSD p-tau181, and MSD p-tau217, but not Simoa p-tau231, were significantly associated with higher WMH volume and lower WM microstructural integrity in 2 WM tracts used as regions of interest (i.e., lower FA values in the genus of corpus callosum and in the hippocampal cingulum bundle). In a sensitivity analysis conducted on 164 participants with all 4 p-tau biomarkers, also Simoa p-tau231 levels were significantly associated with higher WMH volume and lower FA values in the same WM regions of interest.

### Detailed results of “N” biomarkers

A total of 17 studies evaluated the associations between either NfL or t-tau with brain MRI parameters. Studies that used structural MRI techniques showed that NfL levels were negatively correlated with hippocampal volume [26, 65, 66], parietal GM volume [67], ratio of hippocampal volume/TIV [68], right lateral temporal lobe, right inferior parietal, and left superior frontal lobe volumes [69]. In these studies, the associations were mainly found among patients with AD dementia, while no associations were found in potentially pre-symptomatic stages, such as SCD [70]. In the study by Pereira et al. [71], instead, a negative correlation was found between plasma NfL values and hippocampal and nucleus accumbens volumes in MCI patients, but not in either the HC or the AD dementia subsamples, independently of Aβ positivity status. Moreover, Benedet et al. [72] found that increases in plasma NfL levels were associated with reduced GM and WM volumes in both HC and cognitively impaired (CI) older adults. However, more widespread associations were observed in the CI group, with a much larger involvement of frontal and lateral temporal cortices additionally to the medial temporal lobe, when compared with the HC group. Additionally, a study that focused on GM microstructural parameters (by using neurite orientation dispersion and density imaging) found that NfL levels were negatively associated with microstructural integrity of one hippocampal subfield (i.e., the CA4-dentate gyrus) across the AD continuum [59]. In the AD dementia subgroup, instead, negative associations were found between t-tau levels and microstructural integrity of the subiculum and of the CA4-DG.

Associations between BBM of neurodegeneration and WM integrity parameters were also extensively observed. In MCI patients, plasma NfL levels were found to be associated negatively with FA and positively with RD, AxD, and MD values in WM tracts that differed between ApoE ε4 carriers (i.e., widespread across anterior corona radiata, internal capsule and genu of the corpus callosum) and non-carriers (i.e., primarily in fornix, cingulum and uncinate fasciculi) [73]. A positive association between NfL levels and MD values was also found by another study on a sample including patients with MCI and AD dementia in areas of the temporal lobes and the cingulate cortex [74]. Moreover, in a sample of autosomal dominant mutation carriers, a strong association between higher NfL levels and lower FA and higher MD, AxD and RD values across all WM tracts was found, whereas no association were observed in HC [75]. Similarly, other authors who evaluated autosomal dominant mutation carriers described a negative correlation between plasma NfL values and whole brain volume and hippocampal volume, while a positive correlation was found with ventricular volume [76]. In a sample comprehensive of HC, MCI and AD dementia cases, Pereira et al. [71] found a negative correlation between higher plasma NfL and lower left precuneus and right middle temporal gyrus cortical thickness values. After stratifying the sample by diagnosis, no correlation emerged in the HC groups, whereas negative correlations were found in the precuneus in the MCI and AD dementia groups with evidence of Aβ positivity from CSF analysis. In contrast, Elahi et al. [77] found an association between NfL and WMH volume only.

Plasma t-tau was found negatively correlated with mCT in both Aβ-PET positive and Aβ-PET negative participants in a mixed group comprehensive of HC, MCI and AD dementia [57]. This finding was confirmed by Marks and colleagues [66] who observed an association between elevated plasma t-tau levels and lower thickness of temporal cortices in both HC and MCI patients. However, no associations were found between plasma t-tau and FA values in the corpus callosum. In contrast, Illan-Gala et al. [69] found no correlation between plasma t-tau levels and cortical thickness in an AD dementia group. One study found that higher t-tau levels were associated with higher ventricular volume, but not with hippocampal volume in the adjusted analysis [26].

### Detailed results of “X” biomarkers

Of the 5 studies that focused on inflammation markers (i.e. GFAP), 4 assessed the association with structural MRI parameters and one measured the association with metabolites by means of MRS. Ebenau et al. [70] found that higher GFAP levels correlated with lower hippocampal volume and higher MTA in a group of SCD. However, this association was weak and did not survive statistical adjustment for age and sex. Elahi et al. [77], instead, found a significant association with higher WMH volume, but not with global GM volume across the AD continuum. Moreover, other authors showed associations between higher plasma GFAP levels and lower WM volumes in temporal and parietal areas in a mixed group [67], lower temporal cortical thickness and greater WMH volume in Aβ-PET positive HC and MCI patients [78]. One MRS study found that plasma GFAP levels were significantly associated with myo-inositol (mIns) values in the posterior cingulate cortex of HC and MCI patients [53]. Moreover, after stratifying the sample according to ApoE genotype, plasma GFAP levels were significantly associated with the ratio between mIns and total creatine in ApoE ε4 carriers only.

### Quality assessment results

Although variable levels of quality were observed across studies, the overall level appeared to be good, with only 3 out of 33 studies achieving a total score below 50% (Table S2). All studies reported enough details on BBM analysis methods and all but one study reported comprehensive demographic characteristics of the included participant groups. However, only in 13 studies the underlying hypotheses were stated explicitly. No significant difference in quality scores (*t*(31) = − 0.10, *p* = 0.992) was observed between studies that focused on a single blood biomarker only (*n* = 25, 61.1% ± 11.1%) and those that investigated 2 or more biomarkers (*n* = 8, 61.6% ± 4.9%).

## Discussion

This systematic review includes 33 studies that assessed the association between the main BBM and MRI markers in the AD continuum, highlighting consistent associations between these 2 types of marker, in some instances even in the earliest stages of disease (i.e. SCD and MCI). Most of the studies (*n* = 17) focused on markers of neurodegeneration, showing that high levels of NfL are associated with temporal (e.g. hippocampus), frontal and parietal atrophy [67–69]. Moreover, high levels of NfL, as well as t-tau, also correlated with white matter microstructural alterations across different WM tracts [59, 73–76]. Together, these findings suggest that plasma NfL is a promising biomarker that detects neuronal injury in AD, and may have potential for prognosis and monitoring of disease progression. However, in most studies, BBM–MRI associations were only found in a more advanced stage of disease (i.e. in patients with dementia due to AD), and only few of these findings were confirmed in earlier preclinical or prodromal disease stages, i.e. in older adults who were either cognitively unimpaired, had SCD or were experiencing MCI [71, 72]. Since high plasma NfL concentrations are also found in other NDDs [28], further evidence is needed to demonstrate whether plasma NfL concentrations increase already in preclinical and prodromal stages of AD and whether such alterations may be indicative of early neural changes specific for AD. The findings of two studies involving carriers of AD mutations in amyloid precursor protein and in presenilin 1 and 2 genes seem to support this hypothesis [75, 76].

T-tau plasma level increases are also indicative of neurodegeneration across the AD continuum, although this biomarker has been assessed only by a few studies. Negative associations have been found primarily between higher t-tau values and general indices of brain parenchymal loss, e.g. lower mCT and higher ventricular volume, but not with hippocampal volume [79]. These associations were found consistently across studies but one that investigated a sample of people with AD dementia [69]. However, Marks et al. [66] have also found that higher t-tau was associated with reduced temporal cortical thickness in HC and MCI group, but only in one of the two cohorts investigated. More robust evidence is therefore needed to establish how useful plasma t-tau might be in clinical application.

Although the majority of the reviewed studies focused on biomarkers of neurodegeneration, different indices of plasma Aβ were also found associated with MRI metrics. Plasma Aβ42/Aβ40 ratio was negatively associated with MTA in AD dementia patients [50], but also with an index of hippocampal atrophy (i.e., 1 − hippocampal volume/TIV) in older adults with SCD [54]. These findings are in line with previous evidence suggesting that the Aβ42/Aβ40 ratio of these markers derived from plasma may be more useful than individual Aβ peptide concentrations in detecting abnormal Aβ status in both cognitively impaired and cognitively unimpaired participants [10–12]. Moreover, research with older adults with SCD found that higher levels of Aβ42 were associated with lower volume of the left dentate gyrus [33], while higher plasma Aβ40 levels were associated with reduced WM integrity [60]. These findings suggest that the detection of such BBM-MRI associations may signal incipient AD-related pathology and that they may be equally informative in both Aβ positive and negative individuals [57] and across the clinical AD continuum [58].

When assessing the associations between p-tau isoforms and brain MRI parameters, a range of different results was found. While the study by Krebs et al. [54] found no association between plasma levels of p-tau181 and brain MRI outcome measures in SCD, other investigations found that increased levels of this BBM were associated with several indices of GM loss, such as hippocampal volume, reduced total brain and temporo-parietal volumes and temporal cortical thickness, even in HC and MCI groups [51, 52, 62, 64]. Similarly, plasma levels of p-tau isoforms were also associated with WM alterations, i.e., higher WMH volume [51] and decreased microstructural integrity across different WM tracts in various disease stages [51, 63]. Among all these studies, the most consistent association in HC and MCI groups was that between p-tau isoforms (p-tau181 and p-tau217) and temporal grey matter volumes and cortical thickness. This pattern of findings is interesting and, if validated in future studies, alterations in plasma p-tau levels might be a reliable marker of neural alterations due to AD even in preclinical stages.

Increasing evidence suggests that blood GFAP levels can be used to detect early-stage AD [29]. The majority of the studies that focused on this inflammation marker found associations between high levels of GFAP and lower WM in temporal and parietal areas [67], lower temporal cortical thickness and WMH only in Aβ-PET positive cases [78], and altered levels of mIns, a marker of astrocytic function, in a brain area particularly affected by AD, i.e., the PCC/precuneus [53]. Interestingly, these studies describe associations between GFAP and imaging markers in similar brain areas (i.e. temporal and parietal regions) in HC and MCI groups, even when different MRI techniques are used (i.e. structural MRI, DTI or MRS). However, further multimodal imaging studies with different MRI sequences, applied in populations ranging from cognitively unimpaired older adults to the AD spectrum, are needed to confirm these encouraging findings.

Interesting results emerged when associations between BBM and MRI markers were assessed while accounting for Aβ status. In general, associations were detected primarily in Aβ positive older adults only: (1) NfL levels were negatively correlated with cortical thickness in the precuneus of CSF Aβ positive patients with either MCI or AD dementia [71]; (2) higher GFAP levels were associated with lower temporal cortical thickness and greater WMH volume in Aβ-PET positive cases [78]; and (3) higher p-tau 217 and p-tau 181 values were associated with lower temporo-parietal GM volume in Aβ-PET positive cases [64]. However, one study also found that Aβ42 levels were negatively associated with the mCT in Aβ-PET negative cases only [57]. All together, these results suggest a relevant impact of Aβ status, i.e., BBM-MRI associations may be detected primarily in people showing signs of AD pathological changes.

Similarly, stratifying samples by ApoE genotype revealed that several significant associations were detectable in ε4 carriers only: (1) increases in plasma NfL associated with reduced GM volume in HC [72]; (2) Aβ40 level negatively associated with hippocampal volume [55]; and (3) higher GFAP levels positively correlated with mIns/tCr concentration [53]. ApoE genotype, therefore, is confirmed as a strong determinant of AD-related neural alterations and genetic profiling may enhance the detection of clinically relevant BBM–MRI associations, especially in individuals at higher risk of AD.

In some studies, the association between BBM e MRI characteristics was assessed by stratifying by diagnostic group [62, 71]. These investigations highlighted that, in HC and MCI groups, higher p-tau181 levels are associated with lower hippocampal volume and higher NfL values with reduced cortical thickness, hippocampal and accumbens volumes.

Only Pereira et al. [71] investigated how both plasma and CSF NfL concentrations were associated with MRI markers in the same sample: while negative correlations were found between plasma NfL and cortical thickness starting from the MCI stage, negative correlations emerged between CSF NfL and cortical thickness already in HC. These findings suggest that CSF NfL analysis may be more sensitive than blood analysis in detecting AD-related brain atrophy in pre-symptomatic stages. However, these associations were observed for patients with and without amyloid pathology, confirming that NfL is a non-specific marker of AD.

The literature currently available is not exempt from limitations. First, many of the papers included used data obtained from the same datasets: 9 studies used ADNI [26, 52, 58, 62, 63, 71–73, 79]; 4 used BIOFINDER [52, 53, 61, 79]; 3 used the Mayo Clinic Study of Aging cohort [51, 66, 78]; and 2 used the Translational Biomarkers in Aging and Dementia dataset [61, 72]. Although it is not possible to determine the extent of sample overlaps across studies, it is highly likely that the same data from the same participants have been re-used in multiple investigations. As a consequence, this might have introduced a bias in this modestly sized literature, especially for tau and NfL markers. Second, none of the studies had carried out an a priori power calculation and 6 studies included small samples of participants (i.e. *n* < 20). This may be primarily explained by the fact that investigations of BBM for AD have only started in recent years. Indeed, most studies included in this review were exploratory and presented no research hypotheses (third limitation). Fourth, a high degree of heterogeneity was observed in the range of neuroimaging outcome measures investigated, as these were primarily volumes of specific regions of interest. Although focusing on specific brain areas, e.g. the hippocampus, is justified by established knowledge of the typical AD pattern of GM atrophy, this approach might miss clinically relevant associations beyond the medio-temporal lobe and across networks of interconnected brain areas. Fifth, studies of associations of BBM with functional and multimodal MRI investigations are lacking, thus preventing any possible speculations on the potential association between BBM and alterations in brain activity, rather than just structural damage, that may be more sensitive to AD pathology in both preclinical (i.e. cognitively unimpaired older adults) and prodromal (i.e. MCI) stages [80, 81]. Sixth, although the majority of studies (*n* = 24) used Simoa to measure BBM, other analytical methods (e.g., ELISA, MSD, and Luminex) were also used across studies, thus limiting comparability of findings. In this regard, Mielke et al. [51] measured plasma p-tau181 with both Simoa and MSD techniques, reporting a Spearman correlation coefficient of 0.66 indicative of a moderately strong correlation between the two measures. Finally, the different statistical approaches applied in each study could also be a source of heterogeneity in the summarized findings. Indeed, some studies used simple (either parametric or non-parametric) tests to investigate linear correlations between continuous BBM and MRI variables of interest (e.g., [65]), while others applied more refined methods, such as either partial correlation or regression analysis adjusted for multiple factors [51].

## Conclusions

The findings of this systematic review highlight a high degree of association between BBM and a variety of brain MRI outcome measures. Variance in plasma levels of Aβ42 e Aβ42/Aβ40 and higher levels of the other biomarkers (i.e., p-tau, t-tau, NfL and GFAP) were consistently associated with more severe neural alterations. A number of relationships appear early in the course of the disease (even in preclinical stages), suggesting that BBM may represent complementary screening tools for AD. However, given the mild degree of heterogeneity observed in findings in the early preclinical and prodromal stages of the AD continuum, further studies are needed to elucidate how different BBM may be optimally informative of neural alterations in preclinical AD (e.g. HC and SCD with and without evidence of amyloid pathological changes). Moreover, multiple factors can interact or modify the association between BBM and MRI findings, such as age, gender, education, creatinine level, ApoE genotype, Aβ status, thus highlighting the need to consider these variables when assessing BBM-MRI marker relationships but, more in general, when using BBM for clinical purposes. Among the assessed BBM, p-tau isoforms (representative of “T” in the ATN system) are known to be predictive of Aβ status (indicative of “A”) [82] and, according to our results, are consistently associated, from an early clinical stage (i.e. MCI), with temporal grey matter volumes and alterations in cortical thickness (representative of “N”). For this reason, they may be more useful than other BBM in supporting the diagnostic process. The results from this review are encouraging and supportive of further investigations into the combination of MRI and BBM for improving accuracy of early diagnosis, prognosis, and monitoring of disease progression or response to treatment. Future investigations of multimodal neuroimaging outcome measures by means of advanced statistical modelling approaches would be needed to confirm if and to what extent BBM could be indicative of the status of brain alterations across different disease stages.

## Supplementary Information

Below is the link to the electronic supplementary material.

Supplementary file1 (DOCX 1059 KB)
