# Supplementary material for: Association between blood-based protein biomarkers and brain MRI in the Alzheimer’s disease continuum: a systematic review
Source: J Neurol. 2024 Sep 12;271(11):7120–40. doi: 10.1007/s00415-024-12674-w (PMC11560990; doi:10.1007/s00415-024-12674-w)
Supplement: Supplementary file 1 — Supplementary file1 (DOCX 1059 KB) [file 415_2024_12674_MOESM1_ESM.docx]

**Table S1.** Quality assessment criteria.

| **Criteria** | **Score** |
| --- | --- |
| **1 - Case definition** |  |
| 1.1 - Diagnosis (SCD, MCI or AD) | 2: Based on clinical criteria and supported by non-blood biomarkers (i.e. CSF and/or PET)  1: Based on clinical criteria  0: Anamnestic information/medical record |
| 1.2 - Cognitively unimpaired group definition (CU with no SCD) | 2: Based on cognitive assessment and negative non-blood biomarkers (i.e. CSF and/or PET)  1: Based on cognitive assessment  0: Self-reported |
| **2 – General methods** |  |
| 2.1 - *A priori* hypotheses | 1: Clearly stated  0: Not mentioned |
| 2.2 - Demographic data | 1: Comprehensive data reported for all groups  0: Data lacking for one or more groups |
| 2.3 - Inclusion/exclusion criteria | 1: Reasonable and appropriate, considering the possible affecting factors  0: Not reasonable and appropriate |
| 2.4 - Sample size (per diagnostic group) | 2: Power calculation justified  1: *n* ≥ 20  0: *n* < 20 |
| 2.5 - Statistical analyses | 1: Checks and controlling for covariates  0: No checks and/or no controlling for covariates |
| **3 – Blood biomarkers** |  |
| 3.1 - Blood sample collection and pre-processing procedures | 1: Detailed description  0: Lack of details |
| 3.2 - Software, technology and platform used for biomarker quantification | 1: Detailed description  0: Lack of details |
| **4 – MRI measures** |  |
| 4.1 - MRI field strength | 1: 3T or more  0: 1.5T |
| 4.2 - Software, toolkits and (for ROI-based studies) atlases used for image pre-processing and analysis | 1: Detailed description  0: Lack of details |
| 4.3 - Use of correction for multiple comparisons (where relevant, i.e. voxel-based studies or those using multiple ROIs) | 1: Correction applied  0: Correction not applied  *[If not applicable, add an “*” to the final total score]* |
| **5 – Findings/reporting** |  |
| 5.1 - Reporting of results | 1: Detailed description (all studies: sign of statistical association and significance value; ROI-based studies: type of association parameter and confidence interval)  0: Lack of details and/or incongruencies between methods and results |
| 5.2 - Reporting of MRI results | 1: Detailed description (voxel-based studies: peaks, coordinates, cluster extent; ROI-based studies: brain region and side)  0: Lack of details (e.g. only global volumes in ROI-based studies) |
| 5.3 - Limitations | 1: Clearly stated  0: Not mentioned |

AD: Alzheimer’s disease, CSF: Cerebrospinal fluid, CU: Cognitively unimpaired, MCI: Mild cognitive impairment, PET: Positron emission tomography, ROI: Region of interest, SCD: Subjective cognitive decline, T: Tesla

**Score range:**

Minimum score: 0 (lowest quality/highest risk of bias)

Maximum score: 18 (highest quality/lowest risk of bias)

*An asterisk (*) is added to the total quality score for papers that should not be assessed on point 4.3 because not relevant to their methodology.*

**Table S2.** Quality assessment results.

| **Study** | **1.1** | **1.2** | **2.1** | **2.2** | **2.3** | **2.4** | **2.5** | **3.1** | **3.2** | **4.1** | **4.2** | **4.3** | **5.1** | **5.2** | **5.3** | **Total** | **Total (%)^a^** |
| --- | --- | --- | --- | --- | --- | --- | --- | --- | --- | --- | --- | --- | --- | --- | --- | --- | --- |
| **Altomare et al. (2023)** | 2 | 0 | 0 | 1 | 1 | 1 | 0 | 1 | 1 | 1 | 0 | * | 1 | 0 | 1 | 10* | 58.8 |
| **Asken et al. (2022)** | 1 | 1 | 1 | 1 | 0 | 0 | 1 | 1 | 1 | 1 | 1 | 0 | 1 | 0 | 1 | 11 | 61.1 |
| **Barker et al. (2021)** | 1 | 1 | 0 | 1 | 0 | 1 | 1 | 1 | 1 | 1 | 0 | * | 0 | 0 | 1 | 9* | 52.9 |
| **Benedet et al. (2020)** | 1 | 1 | 1 | 1 | 1 | 1 | 1 | 1 | 1 | 1 | 1 | 1 | 1 | 0 | 1 | 14 | 77.8 |
| **Cantero et al. (2016)** | 1 | 1 | 1 | 1 | 1 | 1 | 1 | 1 | 1 | 1 | 1 | 1 | 0 | 0 | 1 | 13 | 72.2 |
| **Cavedo et al. (2020)** | 2 | 0 | 0 | 1 | 1 | 1 | 1 | 1 | 1 | 1 | 1 | 1 | 1 | 1 | 1 | 14 | 77.8 |
| **Ebenau et al. (2022)** | 2 | 0 | 0 | 1 | 1 | 1 | 1 | 1 | 1 | 0 | 1 | * | 0 | 0 | 1 | 10* | 58.8 |
| **Elahi et al. (2020)** | 2 | 2 | 0 | 1 | 0 | 1 | 1 | 1 | 1 | 0 | 0 | * | 0 | 0 | 1 | 10* | 58.8 |
| **Fan et al. (2018)** | 1 | 1 | 0 | 1 | 1 | 1 | 1 | 1 | 1 | 0 | 0 | * | 0 | 0 | 1 | 9* | 52.9 |
| **Gurol et al. (2006)** | 1 | 0 | 0 | 1 | 0 | 0 | 1 | 1 | 1 | 0 | 1 | * | 0 | 0 | 1 | 7* | 41.2 |
| **Hanon et al. (2018)** | 1 | 0 | 0 | 1 | 1 | 1 | 1 | 1 | 1 | 0 | 0 | * | 1 | 0 | 1 | 9* | 52.9 |
| **Hsu et al. (2017)** | 1 | 1 | 0 | 1 | 1 | 1 | 1 | 1 | 1 | 1 | 0 | 0 | 0 | 0 | 1 | 10 | 55.6 |
| **Illan-Gala et al. (2021)** | 1 | 1 | 1 | 1 | 0 | 1 | 1 | 1 | 1 | 1 | 1 | 1 | 0 | 0 | 1 | 12 | 66.7 |
| **Karikari et al. (2020)** | 2 | 2 | 0 | 1 | 1 | 0 | 1 | 1 | 1 | 1 | 0 | 1 | 0 | 0 | 1 | 12 | 66.7 |
| **Karikari et al. (2021)** | 1 | 1 | 0 | 1 | 0 | 1 | 1 | 1 | 1 | 0 | 1 | 0 | 1 | 0 | 1 | 10 | 55.6 |
| **Krebs et al. (2023)** | 0 | 0 | 1 | 1 | 1 | 1 | 1 | 1 | 1 | 1 | 1 | 1 | 1 | 0 | 1 | 12 | 66.7 |
| **Marks et al. (2021)** | 1 | 1 | 0 | 1 | 0 | 1 | 1 | 1 | 1 | 1 | 1 | 0 | 1 | 0 | 1 | 11 | 61.1 |
| **Mattson et al. (2016)** | 1 | 1 | 1 | 1 | 1 | 1 | 1 | 1 | 1 | 0 | 0 | * | 1 | 0 | 1 | 11* | 64.7 |
| **Mattson et al. (2017)** | 1 | 1 | 1 | 1 | 1 | 1 | 1 | 1 | 1 | 0 | 1 | 0 | 0 | 0 | 1 | 11 | 61.1 |
| **Mielke et al. (2021)** | 1 | 1 | 0 | 1 | 1 | 0 | 1 | 1 | 1 | 1 | 0 | 0 | 1 | 0 | 1 | 10 | 55.6 |
| **Nabizadeh et al. (2022a)** | 1 | 0 | 0 | 1 | 0 | 1 | 0 | 1 | 1 | 0 | 1 | 1 | 1 | 1 | 0 | 9 | 50.0 |
| **Nabizadeh et al. (2022b)** | 1 | 1 | 0 | 1 | 1 | 1 | 1 | 1 | 1 | 0 | 1 | 1 | 0 | 0 | 0 | 10 | 55.6 |
| **Ossenkoppele et al. (2021)** | 1 | 1 | 1 | 1 | 1 | 1 | 1 | 1 | 1 | 1 | 1 | 0 | 0 | 0 | 1 | 12 | 66.7 |
| **Parbo et al. (2020)** | 1 | 0 | 0 | 0 | 0 | 1 | 0 | 1 | 1 | 1 | 1 | 1 | 0 | 0 | 1 | 8 | 44.4 |
| **Pereira et al. (2017)** | 1 | 1 | 1 | 1 | 1 | 1 | 1 | 1 | 1 | 0 | 1 | 1 | 1 | 1 | 1 | 14 | 77.8 |
| **Pojak et a. (2016)** | 1 | 1 | 0 | 1 | 1 | 1 | 1 | 1 | 1 | 1 | 1 | 0 | 1 | 0 | 1 | 12 | 66.7 |
| **Rajan et al. (2020)** | 1 | 1 | 0 | 1 | 1 | 1 | 1 | 1 | 1 | 0 | 1 | 0 | 0 | 0 | 1 | 10 | 55.6 |
| **Schultz et al. (2020)** | 1 | 1 | 1 | 1 | 1 | 1 | 1 | 1 | 1 | 1 | 1 | 1 | 1 | 1 | 1 | 15 | 83.3 |
| **Shahid et al. (2022)** | 1 | 1 | 1 | 1 | 1 | 0 | 1 | 1 | 1 | 1 | 1 | 1 | 0 | 0 | 1 | 12 | 66.7 |
| **Shir et al. (2022)** | 1 | 1 | 0 | 1 | 1 | 1 | 1 | 1 | 1 | 1 | 1 | * | 0 | 0 | 1 | 11* | 64.7 |
| **Sotolongo-Grau et al. (2014)** | 1 | 1 | 0 | 1 | 0 | 0 | 1 | 1 | 1 | 0 | 1 | 0 | 0 | 0 | 1 | 8 | 44.4 |
| **Spotorno et al. (2022)** | 1 | 1 | 0 | 1 | 1 | 1 | 1 | 1 | 1 | 1 | 1 | * | 0 | 0 | 1 | 11* | 64.7 |
| **Thijssen et al. (2021)** | 1 | 1 | 0 | 1 | 1 | 1 | 1 | 1 | 1 | 1 | 1 | 1 | 1 | 1 | 1 | 14 | 77.8 |
| **Wang et al. (2021)** | 1 | 0 | 1 | 1 | 1 | 1 | 1 | 1 | 1 | 1 | 1 | 1 | 0 | 0 | 1 | 12 | 66.7 |
| **Weston et al. (2017)** | 1 | 0 | 1 | 1 | 1 | 1 | 1 | 1 | 1 | 1 | 1 | 0 | 0 | 0 | 1 | 11 | 61.1 |

^a^ The total percentage score for those studies not assessed on criterion 4.3 was calculated based on a maximum score of 17.


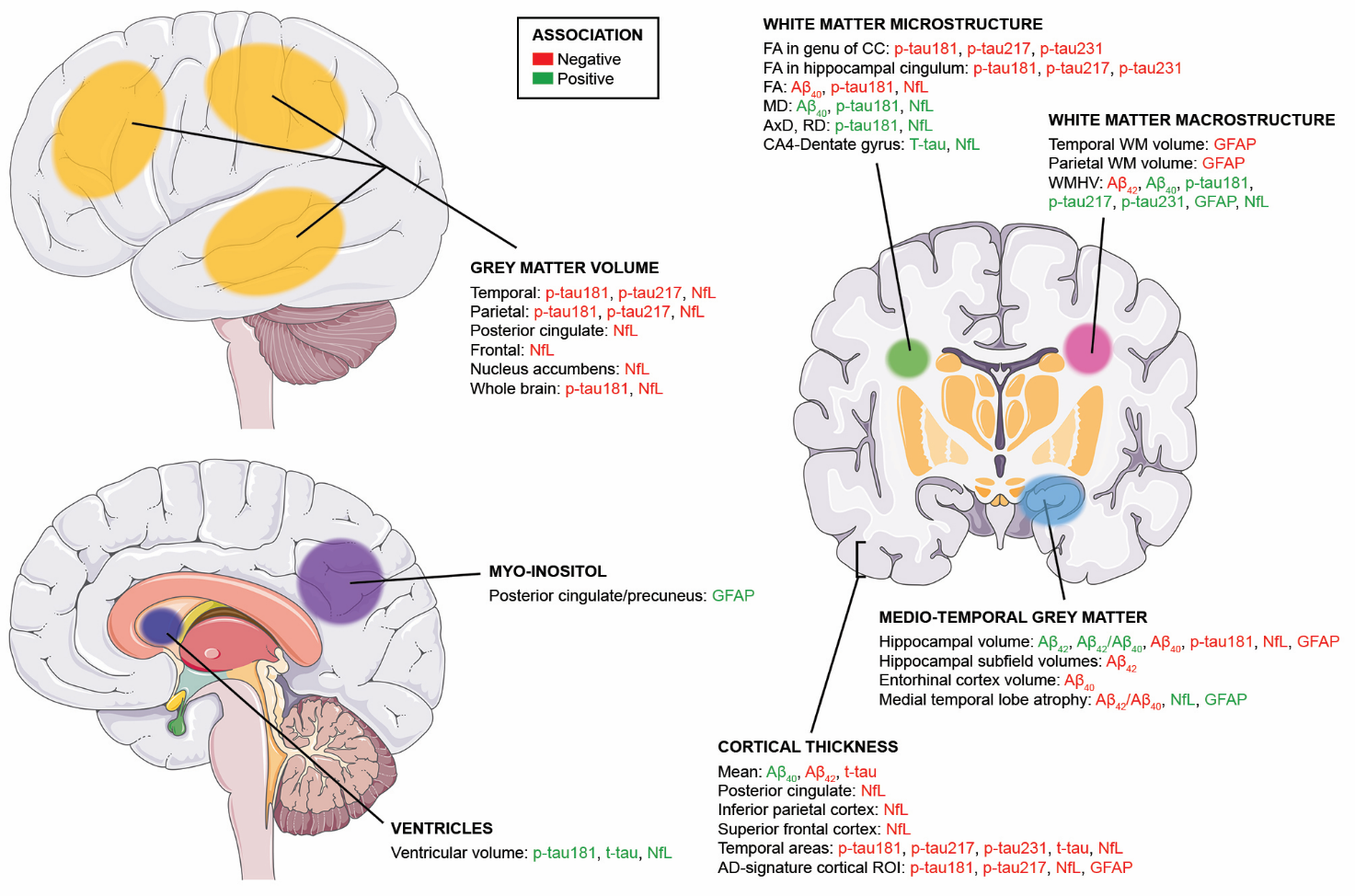


**Fig. S1.** Graphical representation of the associations found between blood-based biomarkers and MRI parameters.
